# Natural Killer Cell Therapy: A New Treatment Paradigm for Solid Tumors

**DOI:** 10.3390/cancers11101534

**Published:** 2019-10-11

**Authors:** Sooyeon Oh, Joo-Ho Lee, KyuBum Kwack, Sang-Woon Choi

**Affiliations:** 1 Chaum Life Center, CHA University School of Medicine, Seoul 06062, Korea; ohsooyoun@hotmail.com (S.O.); sangwoon.choi@gmail.com (S.-W.C.); 2 Graduate school of Internal Medicine, Seoul National University College of Medicine, Seoul 03080, Korea; 3 Department of Gastroenterology and Hepatology, CHA Bundang Medical Center, CHA University School of Medicine, Seongnam 13496, Korea; piolee2000@naver.com; 4 Department of Biomedical Science, College of Life Science, CHA University, Seongnam 13488, Korea; 5 School of Public Health and Health Sciences, University of Massachusetts, Amherst, MA 01003, USA

**Keywords:** immunotherapy, natural killer, autologous, allogeneic, NK cell deficiency

## Abstract

In treatments of solid tumors, adoptive transfer of ex vivo expanded natural killer (NK) cells has dawned as a new paradigm. Compared with cytotoxic T lymphocytes, NK cells take a unique position targeting tumor cells that evade the host immune surveillance by down-regulating self-antigen presentation. Recent findings highlighted that NK cells can even target cancer stem cells. The efficacy of allogeneic NK cells has been widely investigated in the treatment of hematologic malignancies. In solid tumors, both autologous and allogeneic NK cells have demonstrated potential efficacy. In allogeneic NK cell therapy, the mismatch between the killer cell immunoglobulin-like receptor (KIR) and human leukocyte antigen (HLA) can be harnessed to increase the antitumor activity. However, the allogeneic NK cells cause more adverse events and can be rejected by the host immune system after repeated injections. In this regard, the autologous NK cell therapy is safer. This article reviews the published results of clinical trials and discusses strategies to enhance the efficacy of the NK cell therapy. The difference in immunophenotype of the ex vivo expanded NK cells resulted from different culture methods may affect the final efficacy. Furthermore, currently available standard anticancer therapy, molecularly targeted agents, and checkpoint inhibitors may directly or indirectly enhance the efficacy of NK cell therapy. A recent study discovered that NK cell specific genetic defects are closely associated with the tumor immune microenvironment that determines clinical outcomes. This finding warrants future investigations to find the implication of NK cell specific genetic defects in cancer development and treatment, and NK cell deficiency syndrome should be revisited to enhance our understanding. Overall, it is clear that NK cell therapy is safe and promises a new paradigm for the treatment of solid tumors.

## 1. Introduction 

During recent decades, significant strides have been made in the areas of immunology and immunotherapy. Following the success of adjuvant immunotherapy using cytokine induced killer (CIK) cells in the treatment of hepatocellular carcinoma (HCC) [1], natural killer (NK) cell therapy has been discussed as a promising candidate for the next important advance. The existence of NK cells was first suggested in 1964 when it was observed that irradiated mice rejected transplanted bone marrow cells without prior sensitization, depending on histocompatibility [2]. Later, in the 1970s, NK cells were described in more detail when the major histocompatibility complex (MHC) nonrestricted killer cells were found to be naturally cytotoxic to tumor cells, including leukemic cells [3,4,5,6]. After the discovery of NK cells, a great deal of research followed, which elucidated a critical role of NK cells in supporting the whole immune system, identified their association with many human diseases, and even attempted to use NK cells as a form of therapy [7]. Of note, it was demonstrated in more recent studies that NK cells can identify and selectively kill cancer stem cells [8,9,10]. This is particularly important, as it shows that NK cell-based therapy may become an effective method to target quiescent and nonproliferating cancer stem cells to prohibit relapse and metastasis [8]. This suggests that NK cell therapy could be considered as an adjuvant to standard cancer treatment. Currently, NK cell therapy is being discussed as a potential solution to unmet needs, not only in the treatment of cancer, but also that of other human diseases. 

## 2. The Biological Background of NK Cell Therapy

NK cells, an important part of the innate immune system, are characterized by the expression of cluster of differentiation (CD) 56 and the absence of the T cell marker CD3. They comprise 5% to 15% of peripheral blood mononuclear cells (PBMCs), and the population of NK cells is divided into two subsets, CD56^dim^ or CD56^bright^, based on the degree of CD56 fluorescence observed by flow cytometry. More than 90% of NK cells in the peripheral blood are CD56^dim^CD16^+^, while the majority of NK cells in the lymphoid tissues are CD56^bright^CD16^−^ [11,12]. CD56^dim^ NK cells are fully competent cytotoxic cells, while CD56^bright^ NK cells are more suited for cytokine production. Therefore, CD56^dim^ NK cells are considered cytotoxic while CD56^bright^ NK cells are considered immune-regulatory [13]. CD16 is a low affinity Fcγ receptor which confers the ability to recognize antibody-coated target cells and is important for antibody-dependent cell cytotoxicity (ADCC). In this respect, NK cells play a role as effector cells in the adaptive immune system. 

NK recognition of target cells depends on activating and inhibitory receptors expressed on the cell surface. These receptors recognize ligands on target cells. More specifically, the NK cell recognizes the ‘absence’ of a self-antigen through the interaction between its killer cell immunoglobulin-like receptors (KIRs) and MHC class I molecules expressed on the surface of the target cell [14,15]. Inhibitory KIRs are transmembrane molecules coded on chromosome 19 that interact with human leukocyte antigen (HLA)-A, HLA-B, and HLA-C, the MHC class I molecules [16]. When the right MHC class I molecule is recognized by a corresponding KIR, the KIR transmits an inhibitory signal. However, when the MHC class I molecule is missing, no inhibitory signal is generated, and the balance shifts towards activation. This ‘missing-self’ recognition is a vital process for the whole immune system. Cytotoxic T lymphocytes (CTLs), which perform a task similar to NK cells, recognize diseased cells when the cells present ‘altered’ self-antigens on MHC class I molecules. This process is highly dependent on antigen recognition, and CTLs fail to recognize diseased cells if the presentation of self-antigen in the context of self-MHC is lost. To evade host immune surveillance, some virally infected or malignantly transformed cells downregulate or altogether lose expression of MHC-peptide. This loophole in the adaptive immune system can be closed by NK cell activation, and this difference in recognition mechanisms is why NK cells have garnered significant attention and are expected to fill the holes left by CTLs in CIK cell therapy. 

The interaction between KIR and HLA to determine target cell killing provides the scientific background for allogeneic NK cell therapy. Since tumor recognition by NK cells depends on the absence of self-antigen presentation, cancer cells with low HLA expression, especially leukemic cells, are susceptible to lysis by NK cells [17]. However, tumor cells with high expression of HLA such as lymphomas and some solid tumors tend to be more resistant [17]. If the KIR on an NK cell is mismatched with the HLA of a target cell, the chances of cytolysis rise [18,19,20]. Therefore, allogeneic NK cells with a KIR-HLA mismatch are expected to contribute to the killing of those tumor cells resistant to autologous NK cells. Broadly, KIR genes can be divided into two haplotypes, termed A and B. The A haplotype has a fixed number of KIR genes with several inhibitory KIRs and only one activating KIR. By contrast, the B haplotype has a greater number of genes and more than one activating KIR [21,22]. Therefore, the B haplotype is generally preferred as the donor for allogeneic NK cell therapy. The clinical benefit of allogeneic NK cells with a KIR-HLA mismatch has been actively investigated in the treatment of leukemia with or without hematopoietic stem cell transplantation (HSCT) [23]. 

## 3. Low NK Cell Activity and Cancer Development

Epidemiologic studies have indicated that decreased function of NK cells is related to cancer incidence. In 2000, the association between low NK cell cytotoxicity and development of cancer was demonstrated in a prospective cohort study conducted in a Japanese town including 3625 residents followed for 11 years [24]. Although this study demonstrated that NK cell cytotoxicity is a crucial component in immune surveillance of cancer cells, the chromium-51 release assay, which was used in this study to determine the NK cell cytotoxicity, could not be widely utilized in a clinical setting due to the technical difficulties associated with radioactive isotopes. Even without the use of radioactive isotope, a CD107a degranulation assay (another test for the NK cell cytotoxicity) is available, but was considered complicated and time-consuming. Recently, studies with similar aims have been conducted with a novel method. These studies tested the association between cancer incidence and NK cell activity as measured by IFN-γ release upon stimulation. It was shown that lower NK cell activity was associated with increased incidence of cancers in the stomach [25], colorectum [26], and prostate [27,28]. Measuring IFN-γ release to evaluate the functional state of NK cells is easily accomplished with a commercialized kit (NK Vue®, ATGen, Korea) with ‘PROMOCA™ (ATGen, Korea)’, an engineered recombinant cytokine that specifically stimulates NK cells [29]. Introduction of this new test for NK cell function in daily clinical practice increased social awareness that immunity matters in cancer prevention and treatment.

Upon unraveling the epidemiologic association between NK cells and cancer [24], research efforts ensued to use NK cells in the treatment of cancer via adoptive cellular transfer. In both in vitro and in vivo studies, NK cells have been shown to mediate direct killing of human cancer cells in acute myeloid leukemia, acute lymphoblastic leukemia, multiple myeloma, glioblastoma, neuroblastoma, Ewing sarcoma, rhabdomyosarcoma, melanoma, and carcinomas of the breast, lung, ovary, colon, pancreas, renal cells, and stomach [17,30,31,32,33,34,35,36].

## 4. NK Cell Deficiency Syndrome (NKDS)

The role and importance of NK cells is the most pronounced in NKDS. NKDS is an isolated immune deficiency of NK cells in which other components of immune system are intact [37]. NKDS is characterized by a consistent and significant reduction in NK cell numbers, cytotoxic activity, or cytokine production [37]. According to differential findings of the named aspects, NKDS is divided into absolute, classical, and functional NKDS [37]. Patients with NKDS usually present severe and recurrent viral infections or atypical infections at adolescence or young adulthood [37]. The absence of NK cell immune surveillance in these patients was linked to increased susceptibility to development of malignant disease [38,39]. The cause of NKDS is attributed to genetic abnormality of NK cells [40,41,42]. In NKDS, NK cells could not be differentiated or modulated to have normal function with cytokine stimulation, which suggested that NKDS is a permanent condition [43,44]. Nevertheless, NKDS is rarely diagnosed. A recent study, however, suggested that genetic abnormality of NK cells may have higher prevalence than expected although the affected individuals do not present severe infectious disease as was described in NKDS. In this study, defects of NK cell specific genes compared with universal genes for immune system were more closely associated with tumor immune microenvironment that determines prognosis, clinical outcomes, and response to immunotherapy [45]. Depletion of NK cells resulted in failed recruitment of dendritic cells to the tumor microenvironment [46,47]. Genetic analysis suggested that NK cells could recruit not only dendritic cells but also 30 other immune cells and that NK cells are the first responders to detect and kill cancer cells and sequentially recruit many other immune cells, shaping tumor microenvironment into a certain pattern [45]. Therefore, it is highly likely that the NK cell specific genetic defect is closely associated with cancer development. Cancer patients with NK cell specific genetic abnormalities or NKDS may benefit from adoptive NK cell therapy with an allogeneic source. 

## 5. Lymphokine Activated Killer (LAK) Cell Therapy: An Early Form of NK Cell Therapy

In the 1980s, autologous LAK cell therapy with concomitant administration of IL-2 was clinically applied in the treatment of advanced solid tumors for patients who were nonresponsive to standard therapy [48,49]. Lymphocytes obtained from cancer patients by repeated leukapheresis were incubated with IL-2 to generate LAK cells, which were administered to the patients in conjunction with IL-2. Clinical remission was observed in 31% (33/106) of the trial group treated with LAK cells and IL-2 and in 15% (7/47) of the control group treated only with IL-2 [48]. Moreover, complete remission (CR) was noted in eight patients, suggesting that autologous LAK cell therapy was an effective anticancer treatment [48]. In 1989, autologous LAK cells with IL-2 were given to 10 HCC patients, tumor regression was observed in two patients, and the rest maintained stable disease (SD) [50]. Furthermore, autologous LAK cells with IL-2 followed by surgical resection of HCC significantly decreased recurrence rate and increased recurrence-free survival [51,52]. A highlight study which demonstrated survival benefit was reported in 1997. In this phase III randomized trial for 174 primary lung carcinoma patients, autologous LAK cells (1–5 × 10^9^ cells/1 shot) with IL-2 were given combined with or after standard cancer therapy every 2–3 months for 2 years, and the 5- and 9-year survival rates of the immunotherapy group were 54.5% and 52% respectively, compared to 33.4% and 24.2% in the control group (*p* < 0.001) [53]. Despite these encouraging results, autologous LAK cell therapy with IL-2 had critical limitations: the concomitant use of IL-2 was repeatedly noted to cause severe side effects including fever, hypotensive syncope, and vascular leak syndrome [48,53], and IL-2 was known to expand not only NK cells but also regulatory T cells (Tregs), potentiating immune suppression by Tregs [54] so that efficacy could not be proven consistently [55,56].

## 6. Ex Vivo Expansion of NK Cells to Increase the Number

To acquire a sufficiently large number of fully functional NK cells, various culture methods were developed [54]. Ex vivo expansion of NK cells into sufficiently large numbers made it possible to omit the use of exogenous cytokines, thus the moniker ‘LAK’ was dropped. Ex vivo expansion of NK cells used not only IL-2 but also combinations of cytokines with or without feeder cells. When feeder cells were used with cytokines, NK cells could be expanded 100- to 40,000-fold in 2–3 weeks [54]. This was a significant advance compared to 10- to 20-fold expansion when single cytokine was used. As feeder cells, many types of cells are employed including PBMCs, antigen presenting cells, modified K562 cells, and Epstein–Barr virus-transformed lymphoblastoid cell lines [54,57]. However, there are safety concerns in using feeder cells that the feeder cells may contaminate the final therapeutic cell product. Therefore, many groups have developed feeder-free culture system using stimulators instead. So far, many groups have developed good manufacturing practice (GMP) compliant and efficient ex vivo expansion systems [54,57]. Biotechnology has advanced to the point where NK cells can be generated directly from CD34^+^ hematopoietic stem cells (HSCs) [58]. Compared with NK cells harvested from PBMCs, NK cells generated directly from HSCs showed lower expression of inhibitory receptors (KIR2DL1, KIR2DL2/3, and KIR3DL1) and higher expression of activating receptors (NKp30, NKp44, and NKp46) [59]. Moreover, these NK cells exhibited cytotoxicity even without prior exposure to IL-2, while NK cells from PBMCs required IL-2 exposure to exhibit cytotoxicity [59]. More recently, a novel strategy to expand highly functional NK cells by using osteoclasts as feeder cells was proposed [9]. Expanded NK cells from cancer patients had decreased cytotoxicity and lower secretion of IFN-γ compared with those from healthy individuals [9] consistent with previous findings showing decreased expansion efficiency and altered cytokine production in cancer patients [60]. This phenomenon was correlated with increased expansion of T cells, for which the addition of anti-CD3 antibody served as an effective solution [9]. Despite these remarkable advances, different types of culture protocols developed by different groups pose a problem for standardization and generate difficulty in ensuring the reproducibility of results [54,61]. It is believed that different culture methods give rise to NK cells with different immunophenotypes. Researchers should move towards establishing a quality standard for the phenotype of manufactured NK cells because the immunophenotype may be a critical component in determining the function and therapeutic efficacy of NK cells [62]. 

## 7. Current NK Cell Therapy in Solid Tumors

In 2004, autologous NK cells expanded ex vivo with an irradiated human feeder cell-line and IL-2 were tested in patients with malignant glioma [63]. The NK cell infusion with IFN-β in nine patients revealed clinical responses [63]. The same year, another study reported SD in one of 12 patients with refractory colorectal cancer or non-small cell lung cancer (NSCLC) who received infusions of autologous NK cells ex vivo expanded and incubated with heat shock protein 70 (HSP70) [64]. HSP70 is a tumor-selective recognition structure, and NK cells incubated with HSP70 demonstrated enhanced cytolytic activity against HSP70 positive tumors in vitro and in vivo [64]. In 2011, a treatment result for autologous NK cell (expanded with irradiated autologous PBMC as feeder cells, IL-2 and OKT3; OKT3 stimulates the PBMC feeder cells) therapy with lymphodepleting but nonmyeloablative chemotherapy with cyclophosphamide and fludarabine was reported [65]. An average of 4.7 × 10^10^ cells was given to patients with metastatic melanoma or renal cell carcinoma, and no clinical responses were observed. The adoptively transferred NK cells persisted from one week to several months, but the persistent NK cells expressed significantly lower levels of NK group 2-member D (NKG2D, an activating receptor) and could not lyse tumor cells in vitro unless they were reactivated with IL-2 [65]. In 2015, a similar but more encouraging observation was reported. In this study, NK cells were expanded with OK432 (OK432 (Picibanil) is a streptococcal preparation [66] that activates NK cells better than in conventional LAK cells [67]), IL-2, and recombinant human fibronectin fragment (FN-CH296, RetroNectin®)-induced T-cells as a stimulator [68]. These expanded autologous NK cells were given to patients with unresectable, locally advanced digestive tract cancers at doses of 0.5 × 10^9^, 1.0 × 10^9^, and 2.0 × 10^9^ cells/injection [68]. Although no clinical responses were observed in this study, cytotoxicity of patients’ peripheral blood increased two-fold [68]. The same group proceeded to a downstream study where immunoglobulin G (IgG) 1 antibody (trastuzumab or cetuximab) was combined with the same doses of autologous NK cells, and this study was published in 2018 [69]. Nine patients with advanced gastric or colorectal cancers received trastuzumab- or cetuximab-based chemotherapy with autologous NK cells [69]. Among six evaluable patients, four patients presented with SD and the other two with progressive disease (PD) [69]. A pilot prospective cohort study published in 2018 treated colon cancer patients (stage IIIa-c) after radical resection with chemotherapy (5-Fu and oxaliplatin according to the National Comprehensive Cancer Network (NCCN)) with or without autologous NK cell therapy [70]. The autologous NK cells were cultured in an anti-CD16 coated flask with IL-2 and OK432. The 5-year progression free survival (PFS, 51.1% vs. 35%, *p* = 0.044) and overall survival (OS) rate (72.5% vs. 51.6%, *p* = 0.037) were significantly higher in the group with NK cell therapy [70]. In 2019, a clinical trial reported the effect of autologous NK cell therapy administered to advanced lung cancer patients [71]. This therapy, called highly activated NK cell immunotherapy, cultured NK cells using an in vitro culture kit that contains membrane chimeric active cellular factors (HANK Bioengineering Co., Ltd, Shenzhen, China) [71]. A total of 13 patients received the NK cell therapy without other treatment. After 3 months, 11 patients (84.6%) were evaluated as SD and two patients (15.4%) as PD [71]. Another study reported in 2019 was conducted in one patient with NSCLC stage IIIb [72]. Autologous NK cells activated with HSP70 and IL-2 were combined with radiochemotherapy and anti-programmed death-1 (PD-1) antibody nivolumab. The patients exhibited an excellent OS that no viable tumor cells or signs of progression were detected up to 33 months after diagnosis [72]. 

Following the successes in hematologic malignancies [23], the safety and feasibility of allogeneic NK cells were also investigated in solid tumors. For HSCT in hematologic malignancies, HLA compatible donor availability only met around 60% of the demands [73]. Therefore, haplotype HSCT was adopted with T cell depletion strategies to avoid graft-versus-host disease (GVHD) [23]. Despite the worrisome anticipation that lack of mature graft T cells may jeopardize immune recovery and graft-versus-leukemia (GVL) effect, GVL effect was still present, and it was thanks to the graft NK cells matured in vivo after HSCT [23,74]. Following researches showed that a mismatch between donor NK cell KIRs and their cognate HLA class I ligands is an important factor to determine the presence of GVL without GVHD and to improve engraftment rates and survival time [75,76]. In a study conducted in 112 high risk acute leukemia patients undergoing haplotype mismatched HSCT, the probability of event-free survival at 5 years was significantly higher in KIR mismatched group compared to KIR matched group (60% vs. <5%, *p* < 0.0005) [77]. Thus, KIR mismatch was identified as a key factor to strengthen the antitumor effect by allogeneic NK cells. Based on this finding, infusion of allogeneic NK cells with a KIR-HLA mismatch has been widely tested as a monotherapy or as an adjuvant therapy after HSCT in hematologic malignancies, and the outcomes were favorable [23]. The early clinical trials of allogeneic NK cell therapy in solid tumors, therefore, strongly resembled those in hematologic malignancies. In 2005, it was reported that haploidentical allogeneic NK cells were safely transferred to patients with metastatic melanoma and renal cell carcinoma [78]. Three studies in advanced solid tumors reported that infusion of allogeneic NK cells followed by HSCT revealed minor signs of clinical response in 2009, 2011, and 2013 [79,80,81]. In another study reported in 2011, infusion of allogeneic NK cells was followed by lymphodepleting preconditioning chemotherapy (cyclophosphamide and fludarabine) with or without total body irradiation, where transient donor chimerism was observed but limited, likely by reconstituting recipient Tregs [82]. Leaving behind the framework of hematologic treatment, an attempt was made to combine allogeneic NK cell therapy with standard chemotherapy for solid tumors. In a study reported in 2010, patients with NSCLC received 1^st^ or 2^nd^ line chemotherapy and 2–4 doses of allogeneic NK cells (0.2–29 × 10^6^ cells/kg/dose) donated by relatives, and premedication with corticosteroids and H_1_-antihistamine was allowed [83]. The results confirmed the safety and suggested potential clinical efficacy [83]. Another study published in 2016 evaluated the safety and efficacy of allogeneic NK cell monotherapy for advanced solid tumors as well as malignant lymphoma [84]. In this study, allogeneic NK cells, named MG 4101 (GC Pharma, Korea), were derived from random healthy unrelated donors and ex vivo expanded with irradiated autologous PBMC feeder cells, IL-2, and OKT3 [84]. MG4101 was tested for tolerability at dose ranges of 1 × 10^6^ cells/kg to 3 × 10^7^ cells/kg. A maximal tolerable dose (MTD) was not determined, but 3 × 10^7^ cells/kg was found to be the maximal feasible dose. Although all the doses tested were well tolerated with grade one or two toxicities, the final cohort with 3 × 10^7^ cells/kg presented the most frequent incidence of adverse events [84]. Patient antibodies specific for donor NK cells were observed more frequently in repeated injections than in a single injection [84]. Of 17 evaluable patients, eight presented SD and nine PD [84]. Signs of immune activation were observed including upregulation of NKG2D on CD8 T cells and increased production of chemokines, whereas counts for Tregs and myeloid derived suppressor cells (MDSCs) as well as transforming growth factor (TGF)-β production decreased [84]. Of note, patients who received NK cells expressing higher numbers of incompatible KIRs had remarkably enhanced survival [84]. On the contrary, there is one study reported in 2018 that could not identify a relationship between treatment response and KIR-HLA mismatch. In this study, haploidentical NK cells were incubated overnight with 500–1000 U/ml IL-2 after depleting T lymphocytes by anti-CD3 antibody-coated paramagnetic particles and enriching NK cells with the CliniMACS CD56 reagent [85]. A combination therapy of the allogeneic NK cells and anti-CD2 antibody 3F8 was given to 35 patients with high-risk neuroblastoma after lymphodepleting preconditioning chemotherapy [85]. The tested NK cell doses ranged <1 × 10^6^ to 50 × 10^6^ cells/kg. Adverse events of grade three hypertension and grade four pneumonitis in one patient precluded reaching an MTD [85]. Among the 35 patients, 10 patients (29%) had clinical response, 17 (47%) no response and eight (23%) PD. Patients receiving >10 × 10^6^ cells/kg had improved PFS (HR 0.36, 95%CI 0.15-0.87, *p* = 0.022) [85]. No relationship between response and KIR-HLA mismatch or FcγRIII receptor polymorphisms was identified, which may have been due to small sample size [85]. In stage IV HCC, effectiveness of irreversible electroporation combined with allogeneic NK cell therapy was reported in 2018. The allogeneic NK cells were expanded using the Human HANK Cell in vitro Preparation Kit (HANK Bioengineering Co., Ltd, Shenzhen, China) that includes the lethally radiated K562-mb15-41BBL stimulatory cells, plasma treatment fluid, lymphocyte culture fluid additives, serum-free medium additives, and cell infusion additives [86]. In this study including 40 patients, increased OS was observed in the combination therapy group compared with irreversible electroporation monotherapy group (10.1 vs. 8.9 months, *p* = 0.0078) [86]. In 2019, a similar study conducted in 40 unresectable primary liver cancer patients reported that patients who received combination therapy exhibited superior median PFS and OS compared to those who received irreversible electroporation monotherapy (PFS 15.1 vs. 10.6 months, *p*  <  0.05; OS 17.9 vs. 23.2 months, *p*  <  0.05) [87]. In this study, the allogeneic NK cells were donated by KIR-HLA mismatching relatives and expanded using the in vitro preparation kit (HANK Bioengineering Co., Ltd, Shenzhen, China) [87]. ‘Off the shelf’ NK cells, the NK-92 cell line, were also tested in solid tumors. In studies reported in 2008 and 2013, ex vivo expanded NK-92 cells were infused as a monotherapy for advanced solid tumor patients. NK cell doses from 1 × 10^8^ cells/m^2^ to 1 × 10^10^ cells/m^2^ were tested [88,89]. Although grade three/four side effects were reported [88], the incidence was rare, and they were generally evaluated to be safe [89]. The dose of 1 × 10^10^ cells/m^2^ was considered the maximum expandable cell dose with the use of an established culture bag system [89]. Three of 15 patients had some antitumor response, including all three patients with lung cancer [89]. One patient developed anti-HLA antibodies, and the persistence of NK-92 cells was confirmed in two patients [89]. 

## 8. Strategies to Enhance the Effect of NK Cell Therapy

In the treatment of solid tumors, both autologous and allogeneic NK cells have demonstrated potential efficacy, and therefore receive equal attention, but it has not yet been determined which is more effective, and each has its pros and cons. In a clinical trial reported in 2017, the efficacy of allogeneic NK cell therapy was directly compared to that of autologous NK cells in recurrent breast cancer [90]. Allogeneic NK cell therapy fully mismatched for KIR and HLA showed better efficacy in terms of tumor response, the number of circulating tumor cells, immune function, and quality of life, with no difference in adverse effects [90]. Moreover, only allogeneic sources should be considered as an option for cancer patients identified with NK cell specific genetic abnormalities or NKDS [43,44,45]. Even so, allogeneic NK cell therapy is known to have several limitations in comparison to autologous NK cell therapy. Allogeneic NK cells cause side effects that increase in severity linearly with cell doses, or require immunosuppressive pretreatments [83,84,85,89]. In addition, the generation of antibodies against transferred allogeneic NK cells potentially limits the possibility and efficacy of repetitive treatments [84,89]. A strategy to overcome these limitations may be developing autologous NK cells which are as effective as the allogeneic. In a xenograft animal model with ovarian cancer tumor grafts, autologous, or allogeneic NK cells expanded with IL-2 and K562 feeder cells expressing IL-21 were infused [62]. In this in vivo study, autologous NK cells were as effective as the allogeneic NK cells, and the phenotype of NK cells was predominantly CD56^superbright^CD16^+^ [62]. The researchers suggested that CD56^bright^ or CD56^superbright^ subsets are superior to CD56^dim^ subsets in antitumor function. A recent study supports this idea, where intratumoral CD56^bright^ NK cells in bladder cancer showed increased cytokine production and cytotoxicity and were associated with improved survival compared to their CD56^dim^ counterparts [91]. It is interesting that this finding is somewhat contrary to the general belief that more mature CD56^dim^ NK cells may be better for antitumor function. Similarly, a study showed that NK cells with a less mature phenotype, NKG2A^+^CD57^-^NKG2C^-^ (CD57^+^ is a marker of terminal differentiation), were associated with protection from leukemia relapse and improved OS [92]. This is also contrary to the conventional knowledge that mature and memory-like NKG2A^−^selfKIR^+^CD57^+^NKG2C^+^ NK cells generated by cytokine stimulation or CMV infection are more efficacious [93,94,95,96,97,98]. Although a unifying explanation for these seemingly contradictory findings is not yet available, recent observations suggest that the phenotype of NK cells determines the function, and CD56^bright^ or CD56^superbright^ NK cells may not only differentiate into mature cytotoxic NK cells, but also secrete enough cytokines to overcome the immunosuppressive tumor microenvironment [99]. Furthermore, those cytokines may induce the differentiation of cancer stem cells limiting their ability to expand and metastasize [100]. Thus, the limitations of autologous NK cell therapy may be overcome by converting patient NK cells into a CD56^bright^ or CD56^superbright^ phenotype, rather than CD56^dim^ [62,99]. In addition, the conventional designation of maturity by the degree of CD56 fluorescence should be reconsidered. The CD56 fluorescence in this context represents the distinct functional status rather than the maturational status. Further studies are warranted to determine the clinical meaning of the final immunophenotypes of the ex vivo expanded NK cells according to different culture methods. In the long run, defining the optimal immunophenotype of therapeutic NK cells in terms of maturation markers, activating/inhibitory receptors and KIR-HLA mismatch will be extremely useful. Some experts say that ex vivo activation may give rise to KIR-HLA mismatching autologous NK cells, because previously silenced, self-reactive, mismatched NK cells could be reactivated. This is thought to be possible because KIR genes on chromosome 19 and HLA genes on chromosome 6 are inherited independently, in other words KIR expression is independent of HLA expression [101], and anergic NK cells with self-reactive KIRs do exist [102,103]. In an analysis of NK cell clones, CD56^bright^ NK cells showed higher cloning efficiency compared with CD56^dim^ or CD57^+^ subsets [104]. Furthermore, it was demonstrated that CD57^+^ subsets could reverse, and acquire a CD57^-^ phenotype in specific conditions, which had previously been considered impossible [104]. These recent discoveries suggest that an era of generating therapeutic NK cells with an optimal immunophenotype may arrive sooner than expected. 

Early trials have been conducted in advanced cancer patients with a high tumor burden. In those studies, signs of clinical response were observed as was indicated by preclinical models that NK cell therapy prevented migration and invasion of cancer cells contributing to prevention of systemic metastasis [36]. However, such response was not dramatic as it did not meet the expectation of the scientific community. The reason for the subdued response was attributed to the immunosuppressive microenvironment that accompanies advanced cancer with a high tumor burden. In severely immunosuppressive microenvironment, adoptively transferred immune cells are easily disengaged, losing their cytotoxic activity and ability to infiltrate into tumor tissues [53]. On the contrary, promising results came from phase III trials conducted in early stage cancer patients, who were left with minimal tumor burden after curative treatment [1,52,53]. In the early stage cancer patients, these studies clearly demonstrated survival benefit of adoptive immune cell therapy with autologous T cells or NK cells. These observations are supported by laboratory findings that NK cells play a more potent role in tumor initiation or prevention, as opposed to slowing tumor growth and progression, in a *KRAS* knock-in mouse lung cancer model [35], and that NK cells could identify and selectively kill quiescent cancer stem cells in many other studies [8]. Based on these findings, it can be inferred that solid tumor patients who would get the maximal benefit from NK cell therapy are likely to be the ones with a low or minimal tumor burden with a purpose to prohibit cancer relapse and metastasis.

For patients with a high tumor burden, combination therapy with standard anticancer therapy seems safe and feasible. Different adjunct treatments were tried along with NK cell therapy. For solid tumors, the use of high-intensity chemotherapy, as was done in hematologic malignancies, may not have enough benefit weighed against the anticipated risk. Immunosuppressive therapy using chemotherapy or corticosteroids may facilitate the survival and function of adopted NK cells, but may have the downside of suppressing the patient’s native immune system. In contrast, combination therapy with IgG antibodies, both as molecularly targeted agents and checkpoint inhibitors, may have potential benefit of utilizing CD16, the low affinity Fcγ receptor on NK cells, coupling NK cell cytotoxicity with ADCC [69]. In in vitro studies, trastuzumab and pertuzumab increased the cytotoxic level of IL-2 activated NK cells targeting ERBB2 positive cancer cell lines [105], and an anti-programmed cell death-ligand 1 (PD-L1) antibody avelumab increased ADCC by NK cells targeting triple negative breast cancer cells [106]. In a preclinical study, activated NK cells, in combination with the anti-GD2 antibody dinutuximab, improved survival of neuroblastoma mice after surgical resection [107]. In addition, avelumab could greatly enhance ADCC of the high-affinity NK cell line in meningioma in vitro and in vivo [108]. In particular, combination therapy with checkpoint inhibitors may have an additional benefit by unleashing exhausted immune cells [72]. In mouse models of cancer, NK cells expressed PD-1 and cancer cells expressed PD-L1, and a PD-1/PD-L1 blockade elicited a strong NK cell response [109]. In a preclinical ovarian cancer model, PM21-particle expanded NK cells had increased antitumor efficacy when combined with an anti-PD-L1 antibody [110]. Amongst multikinase inhibitors, sorafenib and regorafenib are predicted to have a potential synergistic effect when combined with NK cell therapy [111,112,113,114]. To evade host immune surveillance, certain tumor cells such as HCC express a disintegrin and metalloproteases 9 (ADAM9) protease that cleaves membrane bound MHC class I-related chain A (MICA), the ligand for NKG2D. Preclinical studies demonstrated that this MICA shedding can be inhibited by sorafenib and regorafenib [111,112,113,114]. In addition to immune modulatory and antitumor effects, sorafenib and regorafenib inhibit the expression of ADAM9 protease. This can directly restore antitumor NK cell activation and may enhance the efficacy of NK cell therapy.

## 9. Conclusions and Future Perspectives 

Recently, immunological treatments have become a new paradigm in medicine. Immune check-point inhibitors significantly improved the life expectancy of cancer patients. Chimeric antigen receptor (CAR) T cell therapy provided a cure for some patients who were otherwise untreatable [115]. Compared to these dramatic successes, NK cell therapy is not yet satisfactory. There are numerous ongoing clinical trials on NK cell therapy all over the world. In ClinicalTrials.gov, 28 trials for solid tumors and 31 trials for hematologic malignancies were searched (search term: “NK cell” AND “Therapy”; recruitment status: “Recruiting” or “Active, not recruiting”; search date 30th Aug 2019). The search results for solid tumors were listed in Table 1. The trials are in phase I/II testing safety and efficacies as a monotherapy, an adjuvant therapy, and a combination therapy in addition to standard or trial anticancer therapy. The sources of allogeneic NK cells ranged from haploidentical donors, unrelated donors, umbilical cord blood, NK cell lines, to engineered NK cells including CAR-NK cells. 

Since its first introduction to clinical research, NK cell therapy has slowly improved, both in quantity and quality, during the past 30 years. In the early years when ex vivo expansion of NK cells was not available, NK cells were acquired by repetitive leukapheresis, stimulated ex vivo and re-infused to patients with IL-2 in LAK cell therapy. In recent years, many institutes developed GMP compliant culture methods to expand NK cells ex vivo to a sufficiently large number, even from CD34^+^ HSCs. This advancement in biotechnology may accelerate the speed of research on NK cell therapy in the future. Allogeneic NK cell therapy is expected to be incorporated into the standard therapy of hematologic malignancies in the near future [23]. In solid tumors, autologous and allogeneic NK cell therapies both have demonstrated potential efficacy and are getting equal scientific attention. Both have pros and cons. In general, autologous NK cells are discouraged due to their low efficacy, whereas allogeneic NK cells produce more side effects and possess the potential for rejection, precluding repetitive treatments. A recent in vivo study suggested that these problems may be solved by developing autologous NK cells that mirror the allogeneic efficacy, and that the key to doing this is to modulate the final immunophenotype of the NK cell. In addition, currently available molecularly targeted agents and checkpoint inhibitors may directly facilitate the action of adoptively transferred NK cells by acting as a medium for ADCC, helping to overcome the immune exhaustion or inhibiting tumor immune evasion. 

A recent study pointed out that NK cell specific genetic defects may contribute to cancer development and determine treatment outcome. In this regard, revisiting the NKDS and investigating the NK cell specific genetic defects that are subclinical may provide a new perspective in cancer prevention and treatment. Overall, NK cell therapy has been safely administered and demonstrated great potential to enhance clinical outcomes in cancer patients. In the long run, NK cell therapy is expected to provide solutions for unmet medical needs. 

## Figures and Tables

**Table 1:** On-going clinical trials on natural killer (NK) cell therapy for solid tumors.

Identifier	Phase	Status	Start Year	Title	Condition	NK Cell Source	Country	Location
NCT00720785	I	Recruiting	2008	Natural Killer Cells and Bortezomib to Treat Cancer	Chronic Myeloid Leukemia, Multiple Myeloma, Pancreatic/Colon/Rectal/Non-small Cell Lung Cancer	Autologous	USA	National Institutes of Health Clinical Center
NCT01807468	II	Active, not recruiting	2013	Haploidentical Stem Cell Transplantation and NK Cell Therapy in Patients with High-risk Solid Tumors	Neuroblastoma, Ewing Sarcoma, Rhabdomyosarcoma, Osteosarcoma, Soft Tissue Sarcoma	Haploidentical	Republic of Korea	Samsung Medical Center
NCT01857934	II	Recruiting	2013	Therapy for Children with Advanced Stage Neuroblastoma	Neuroblastoma	Allogeneic	USA	St. Jude Children’s Research Hospital
NCT02100891	II	Recruiting	2014	Phase 2 STIR Trial: Haploidentical Transplant and Donor Natural Killer Cells for Solid Tumors	Ewing Sarcoma, Neuroblastoma, Rhabdomyosarcoma, Osteosarcoma, CNS Tumors	Haploidentical	Republic of Korea	Samsung Medical Center
NCT02271711	I	Recruiting	2015	Expanded Natural Killer Cell Infusion in Treating Younger Patients with Recurrent/Refractory Brain Tumors	Medulloblastoma/Ependymoma/Medulloblastoma	Autologous	USA	M.D. Anderson Cancer Center
NCT02370017	II	Recruiting	2015	Combined Effect of Natural Killer Cell and Doublet Chemotherapy in Advanced NSCLC as the 1st Line Treatment	Non-small Cell Lung Cancer	Autologous	Republic of Korea	Daejeon St. Mary’s Hospital
NCT02507154	I/II	Recruiting	2015	Reactivating NK Cells in Treating Refractory Head and Neck Cancer	Nasopharyngeal Cancer, Head and Neck Squamous Cell Carcinoma	Autologous	Singapore	National University Hospital
NCT02409576	I/II	Recruiting	2015	Pilot Study of Expanded, Activated Haploidentical Natural Killer Cell Infusions for Sarcomas	Ewing Sarcoma, Rhabdomyosarcoma	Allogeneic	Singapore	National University Hospital
NCT02465957	II	Active, not recruiting	2015	QUILT-3.009: Patients with Stage IIIB or Stage IV Merkel Cell Carcinoma	Stage IIIB Merkel Cell Carcinoma, Stage IV Merkel Cell Carcinoma	NK-92	USA	Robert H. Lurie Comprehensive Cancer Center and 2 more locations
NCT02734524	II	Recruiting	2016	A Clinical Research of NK Cell Infusion Combined with Chemotherapy in the Treatment of Non-small Cell Lung Cancer	Non-small Cell Lung Cancer	Autologous	China	Southwest Hospital of Third Millitary Medical University
NCT02650648	I	Recruiting	2016	Humanized Anti-GD2 Antibody Hu3F8 and Allogeneic Natural Killer Cells for High-Risk Neuroblastoma	Neuroblastoma	Blood-related and haploidentical	USA	Memorial Sloan Kettering Cancer Center
NCT03242603	I/II	Recruiting	2017	Immunotherapy of Neuroblastoma Patients Using a Combination of Anti-GD2 and NK Cells	Neuroblastoma	Haploidentical	Singapore	National University Hospital
NCT03213964	I	Recruiting	2017	Intraperitoneal Delivery of Adaptive Natural Killer Cells (FATE-NK100) with Intraperitoneal Interleukin-2 in Women with Recurrent Ovarian, Fallopian Tube, and Primary Peritoneal Cancer	Epithelial Ovarian Cancer, Fallopian Tube Cancer, Primary Peritoneal Cancer	Haploidentical or not fully HLA-matched related donor	USA	Xuzhu medical university Masonic Cancer Center
NCT03410368	II	Recruiting	2018	NK Cell-based Immunotherapy as Maintenance Therapy for Small-Cell Lung Cancer.	Small-Cell Lung Cancer	Autologous	China	The First Hospital of Jilin University
NCT03420963	I	Recruiting	2018	Ex-Vivo Expanded Allogeneic NK Cells for The Treatment of Pediatric Solid Tumors	Relapsed or Refractory Solid Tumors	Umbilical cord blood	USA	M.D. Anderson Cancer Center
NCT02573896	I	Recruiting	2018	Immunotherapy of Relapsed Refractory Neuroblastoma with Expanded NK Cells	Neuroblastoma	Autologous	USA	Children’s Hospital Los Angeles and 11 more locations
NCT03619954	I	Recruiting	2018	NK Cells Infusion for Advanced Malignancies	Advanced Malignant Tumors	?	China	Anhui Provincial cancer center
NCT03209869	I	Recruiting	2018	Treatment of Relapsed or Refractory Neuroblastoma with Expanded Haploidentical NK Cells and Hu14.18-IL2	Neuroblastoma	Haploidentical	USA	University of Wisconsin Carbone Cancer Center
NCT03662477	I	Recruiting	2018	Effect of NK Cell Immunotherapy on Advanced Lung Adenocarcinoma Adenocarcinoma with EGFR Mutation	Non-small Cell Lung Cancer	Autologous	China	Shenzhen Luohu Hospital
NCT03319459	I	Recruiting	2018	FATE-NK100 as Monotherapy and in Combination with Monoclonal Antibody in Subjects with Advanced Solid Tumors	Advanced Solid Tumors	Haploidentical or not fully HLA-matched related donor	USA	UCSD Moores Cancer Center and 3 more locations
NCT03634501	I/II	Recruiting	2018	Clinical Study on Antitumor Effect Induced by Activated Primary Natural Killer Cells	Lung Cancer, Breast Cancer, Colon Cancer, Pancreatic Cancer, Ovarian Cancer	Autologous and/or umbilical cord blood	China	Xuanwu Hospital
NCT03882840	I/II	Recruiting	2019	Induced-T Cell Like NK Cellular Immunotherapy for Cancer Lack of MHC-I	Advanced Cancer with Low or No Expression of MHC-I	T-like NK cells (Engineered from patient T cells)	China	The Second Affiliated Hospital of Guangzhou Medical University
NCT03958097	II	Recruiting	2019	A Pilot Study of NK Cell Combined with PD-1 Antibody as Second Line Therapy for Advanced Driver Mutation Negative Non-small Cell Lung Cancer	Non-small Cell Lung Cancer	Autologous	China	The First Hospital of Jilin University
NCT03940820	I/II	Recruiting	2019	Clinical Research of ROBO1 Specific CAR-NK Cells on Patients with Solid Tumors	Solid Tumor	Anti-ROBO1 CAR-NK cells	China	Suzhou Hospital Affiliated to Nanjing Medical University
NCT03941457	I/II	Recruiting	2019	Clinical Research of ROBO1 Specific BiCAR-NK Cells on Patients with Pancreatic Cancer	Pancreatic Cancer	Anti-ROBO1 CAR-NK cells	China	Department of Radiology, Shanghai Ruijin Hospital
NCT03853317	II	Active, not recruiting	2019	QUILT-3.063: A Study of N-803, haNK and Avelumab in Patients with Merkel Cell Carcinoma That Has Progressed after Checkpoint Therapy	Merkel Cell Carcinoma	haNK (an off-the-shelf CD16-targeted NK cells)	USA	Chan Soon-Shiong Institute for Medicine
NCT03941262	I	Recruiting	2019	Autologous Natural Killer Cells in Participants with Pathologically Confirmed Cancer	Refractory Cancer	Autologous	USA	Sarcoma Oncology Research Center
NCT03841110	I	Recruiting	2019	FT500 as Monotherapy and in Combination with Immune Checkpoint Inhibitors in Subjects with Advanced Solid Tumors	Advanced Solid Tumors	FT500 (an off-the-shelf, iPSC-derived NK cells)	USA	UCSD Moores Cancer Center and 2 more locations
